# Emerging Roles of Mitochondrial Ribosomal Proteins in Plant Development

**DOI:** 10.3390/ijms18122595

**Published:** 2017-12-02

**Authors:** Pedro Robles, Víctor Quesada

**Affiliations:** Instituto de Bioingeniería, Universidad Miguel Hernández, Campus de Elche, 03202 Elche, Spain; probles@umh.es

**Keywords:** mitoribosomes, mitochondrial ribosomal proteins (mitoRPs), arabidopsis, ribosomal filter hypothesis, plant development, mutants

## Abstract

Mitochondria are the powerhouse of eukaryotic cells because they are responsible for energy production through the aerobic respiration required for growth and development. These organelles harbour their own genomes and translational apparatus: mitochondrial ribosomes or mitoribosomes. Deficient mitochondrial translation would impair the activity of this organelle, and is expected to severely perturb different biological processes of eukaryotic organisms. In plants, mitoribosomes consist of three rRNA molecules, encoded by the mitochondrial genome, and an undefined set of ribosomal proteins (mitoRPs), encoded by nuclear and organelle genomes. A detailed functional and structural characterisation of the mitochondrial translation apparatus in plants is currently lacking. In some plant species, presence of small gene families of mitoRPs whose members have functionally diverged has led to the proposal of the heterogeneity of the mitoribosomes. This hypothesis supports a dynamic composition of the mitoribosomes. Information on the effects of the impaired function of mitoRPs on plant development is extremely scarce. Nonetheless, several works have recently reported the phenotypic and molecular characterisation of plant mutants affected in mitoRPs that exhibit alterations in specific development aspects, such as embryogenesis, leaf morphogenesis or the formation of reproductive tissues. Some of these results would be in line with the ribosomal filter hypothesis, which proposes that ribosomes, besides being the machinery responsible for performing translation, are also able to regulate gene expression. This review describes the phenotypic effects on plant development displayed by the mutants characterised to date that are defective in genes which encode mitoRPs. The elucidation of plant mitoRPs functions will provide a better understanding of the mechanisms that control organelle gene expression and their contribution to plant growth and morphogenesis.

## 1. Introduction

Ribosomes are the cellular machinery that performs protein synthesis from translating the information contained in mRNA molecules. They are ribonucleoprotein complexes that comprise two subunits, one large (LSU) and one small (SSU), and consist of rRNAs and proteins. In a eukaryotic cell, ribosomes are found in the cytoplasm, mitochondria and plant chloroplasts. In evolutionary terms, chloroplasts and mitochondria derive from the ancestors of current cyanobacteria and α-proteobacteria, respectively, that established a symbiotic relationship with an ancestral eukaryote. During evolution, the number of genes in the endosymbiotic genomes drastically dropped as most were transferred to the nuclear genome. Hence they contain only a few dozen genes in the present-day. Transferred genes also include those that encode mitochondrial and plastid ribosomal proteins, although both organelles have retained in their genomes some genes encoding the ribonucleoprotein complexes.

A mitochondrion is a double-membrane organelle essential for life, and is present virtually in all eukaryotic cells, except for several protozoa, some fungi and mature red blood cells in mammals [1]. Widely known for its role in ATP production through oxidative phosphorylation, the mitochondrion also plays a key role in a wide range of cellular functions, such as fatty acid oxidation, amino acid biosynthesis, apoptosis and transduction of cellular signals [2]. All these processes require accurate protein synthesis inside the organelle.

Mitochondrial ribosomes, or mitoribosomes, are essential for the synthesis of oxidative phosphorylation machinery. They have been subjected to major research efforts in yeast and humans, in the former for being a model system for eukaryotic cell biology, and in the latter for mitoribosomes being implicated in human health. Both the composition and structure of mitoribosomes in both systems have been solved by cryo-EM [3,4]. In contrast, the precise structure and protein composition of plant mitoribosomes are not yet known [5], although they are bigger (around 78S) than mammalian mitoribosomes (55S) [6]. Regarding rRNA composition, plant mitoribosomes are constituted of three different molecules (5S, 18S and 26S), all of which are encoded by the mitochondrial genome [7]. In contrast, the genes that encode plant mitochondrial ribosomal proteins (hereafter mitoRPs) lie in both the nuclear and mitochondrial genomes, and their numbers vary from one species to another. Accordingly, Bonen and Calixte [8] identified in *Arabidopsis thaliana* (hereafter Arabidopsis) and rice nuclear genomes 46 and 48 genes, respectively, that encode mitoRPs were 11 of these genes present in multiple copies (2–4). Furthermore, these authors also identified seven additional mitoRP genes in the Arabidopsis mitochondrial genome. Sormani et al. [9] described 71 genes in Arabidopsis that encode mitoRPs, with 63 and 8 of them located in the nuclear and the mitochondrial genomes, respectively. Similar numbers were also reported for potato and broad bean with 68 to 80 mitoRPs [10,11]. In contrast, a typical eubacteria such as *Escherichia coli* contains 54 ribosomal proteins, 33 and 21 in the LSU and SSU subunits, respectively [12].

In ribosomes, each ribosomal protein type is represented by a single polypeptide. However, as stated above, several ribosomal proteins are encoded by two genes or more of the same family (paralogous genes), which results from gene duplications. In Arabidopsis, 13 plastid ribosomal proteins and 16 mitoRPs are encoded by small-multigenic families [9], whereas the 81 ribosomal protein types that integrate cytoplasmic ribosomes are encoded by 251 genes [13]. The expression patterns of paralogous genes may differ, as shown for members of the families that encode the Arabidopsis cytoplasmic S18, L16 and S15 proteins [14,15,16]. This suggests that they may be involved in different developmental processes and/or may act at distinct times in tissues or cell types. Furthermore, translation in plants may be regulated by modifying the composition of the proteins that form part of the ribosome. Accordingly, the abundance and composition of polysomes (groups of ribosomes that translate the same mRNA) vary while bean leaves grow and develop [17]. In addition, transcript profiling in *Brassica napus* has revealed the existence of functional divergence and expression networks among the paralogous genes that encode ribosomal proteins, which strongly suggests their participation in development, differentiation and/or tissue-specific processes [18].

The presence in plants of small gene families of mitoRPs, whose members are functionally divergent, has also been reported. In line with this, four paralogues of mitochondrial L12 protein in potato have been differentially associated with mitochondrial ribosomes [19] and eight members of the Arabidopsis L18 family have highly divergent sequences and specificities during plant growth and development [20]. This supports the hypothesis of the heterogeneity of plant mitoribosomes, which would allow a highly dynamic mitochondrial translational machinery composition [21], and constitutes the basis of the so-called ribosomal filter hypothesis proposed by Mauro and Edelman [22]. This hypothesis argues that ribosomes are not simply machines that carry out translation, but they are also able to regulate gene expression. Consequently, the ribosome would act as a filter that would select specific mRNA molecules for translation in response to different physiological conditions during development. Hence distinct populations of ribosomes would have varying abilities to translate particular mRNA molecules [5].

This review principally focuses on analysing the perturbed plant developmental processes and the resulting phenotypes hitherto described, caused by mutations in genes that encode mitoRPs, or in other genes that impair the mitoRP function.

## 2. Developmental Defects Caused by Mutations in Genes that Encode mitoRPs

Plant growth, including cell expansion and division, is fundamental for plant development and morphogenesis, and requires a substantial supply of energy and metabolites. This is in consonance with the increased number of mitochondria in cells observed during leaf and reproductive development [21]. Therefore, perturbed mitochondrial translation is expected to severely impair mitochondrial activity and, consequently, plant developmental processes will require this organelle to perform well. To date, mutations in both nuclear and mitochondrial genes that encode mitoRPs have been reported to affect plant growth and development. The phenotypic alterations described to date due to these mutations have clearly shown the involvement of mitoRPs in several aspects of plant development and different plant processes. Accordingly, mutations in some mitoRPs result in an embryo-lethal phenotype while the analysis of other mutants has revealed a role for some mitoRPs in leaf morphogenesis and in reproductive tissue formation (Table 1).

### 2.1. Embryo-Lethal Mutations in mitoRPs

In Arabidopsis, the *hes* (*heart stopper*) mutant, which is affected in mitochondrial ribosomal protein L18, displays a low proliferation of seed endosperm cells and arrested embryo development in the late globular stage (Table 1) [20]. *hes* embryos have been cultured in vitro, but their phenotypic rescue has not yet been achieved. Although some give rise to callus, they do not differentiate into plants despite adding hormones to the culture medium. This indicates that HES is required for cell growth, differentiation and the establishment of organ patterns. Zhang et al. [20] identified eight genes that encode L18 ribosomal proteins in the Arabidopsis nuclear genome, five and two of them potentially located in the mitochondria and chloroplasts, respectively. The subcellular localisation of the remaining one is ambiguous. Interestingly, these authors found that the members of this small gene family markedly differ in their amino acid sequences. Besides, the *hes* mutant phenotype cannot be complemented by other L18 members. *HES* expression is restricted to tissues that undergo active cell division and differentiation, including the embryo and root tip. The spatial expression pattern of *HES* corresponds well to the mutant phenotypes of the *hes* individuals during seed development. In *E. coli*, L18 is an essential protein that forms part of the central protuberance of the 50S subunit of the ribosome and binds to 5S and 23S rRNAs [29]. The 3D modelling of mutant and wild-type L18 proteins suggests that the amino acid substitution present in the *hes* mutant protein might affect its binding to 5S rRNA and hence, the stability of the 50S subunit. However, the *hes* mutation does not alter the mitochondria morphology in the embryo or the endosperm. This made Zhang et al. [20] to propose that the effects on development caused by impaired *HES* function might be due to alterations in the mitochondrial metabolic processes affected by reduced mitochondrial translation, which would require L18. Consistently with this, these authors identified several marker genes of mitochondrial dysfunction, such as *ALTERNATIVE OXIDASE 1a* (*AOX1a*) and *NAD(P)H DEHYDROGENASE* (*NDB4*), which are overexpressed in the *hes* mutant compared with the wild type. They concluded that the strong divergence between the genes that encode L18 proteins, the restricted expression pattern of *HES* and the inability of other L18 proteins to complement the *hes* mutant phenotype all support the existence of heterogeneous mitoribosomes, which would consist in different L18 proteins with distinct functions. Heterogeneous mitoribosomes would likely have different properties and could modulate gene expression, which would affect the translation efficiency of certain types of mRNAs in response to different physiological requirements during development.

Interestingly, the loss of function of one of the Arabidopsis L18 proteins, EMB3105 encoded by the AT1G48350 gene, and putatively localised in plastids, causes embryonic lethality in the same developmental stage as the *hes* mutations does (the globular stage; [30,31]).

### 2.2. Effects of the Mutations in mitoRPs on Reproductive Tissues

The characterisation of plant mutants has revealed a role for some mitoRPs in reproductive tissue formation. Along these lines, the Arabidopsis *huellenlos-1* (*hll-1*) and *hll-2* mutants are good representatives (Table 1) [23]. *hll-1* and *hll-2* individuals carry point mutations which lead to truncated L14 mitoribosomal proteins and cause arrested ovule development before or immediately after the formation of integuments of ovules (*hll-1*), or after integuments have begun to spread around the nucela (*hll-2*) [23]. *hll-1* and *hll-2* also present alterations in the gynoecium, which is smaller than in the wild type and has a few ovules. In the Arabidopsis genome, Skinner et al. [23] identified a paralogous gene functionally related with *HLL*, *HUELLENLOS PARALOG* (*HLP*). The ectopic expression of *HLP* complements the *hll* mutant phenotype [23]. This contrast with the lack of complementation of the *hes* mutant phenotype by other L18 proteins (see above). Notwithstanding, both genes differ in their expression levels in organs because transcripts of the *HLP* and *HLL* genes are detected mostly in pistils and leaves, respectively. In addition, the HLL and HLP proteins fused to the green fluorescent protein (GFP) are targeted to mitochondria, which supports a role for both proteins in this organelle. In *E. coli*, the L14 ribosomal protein is an essential protein that binds to rRNA and participates in forming a bridge between the two ribosomal subunits [32]. This falls in line with the phenotype of gametic lethality found in *hll*. Skinner et al. [23] proposed that the phenotypic effect of *hll* mutations on reproductive development might be explained by carpels and ovules’ considerable energy requirements. In agreement with this, an increase in the number of mitochondria in reproductive tissues and the specific degeneration of ovaries in transgenic plants with reduced activity of the citrate synthase enzyme, commonly used as a quantitative marker of the presence of intact mitochondria, have been reported [33,34].

Karyogamy, this being the fusion of two cellular nuclei to produce a single nucleus, is fundamental for the sexual reproduction of animals and plants [35]. An analysis of an array of Arabidopsis mutants, affected in the fusion of the polar nuclei during female gametophyte development, allowed Portereiko et al. [24] to identify six mutants, namely *nuclear fusion defective 1* (*nfd1*) to *6*. One of these mutants, *nfd1*, is also affected in kariogamy during fertilisation and male gametophyte development (Table 1). Defective kariogamy is due to the non-fusion of outer nuclear membranes [24]. The nuclear *NFD1* gene encodes the L21 mitoRP of Arabidopsis, and the orthologous protein in *E. coli* is a component of the 50S subunit of the mitoribosome, which binds to 23S rRNA [36,37]. The Arabidopsis genome contains a single gene for the mitochondrial L21 protein, which is expressed in all the studied organs. Portereiko et al. [24] proposed that the *nfd1* mutation might impair nuclear fusion by altering the composition of the phospholipids of the nuclear membrane. The importance of mitochondria in kariogamy is further supported by the identification of four additional *nfd* mutants (*nfd3* to *6*) which also carry T-DNA insertions in nuclear genes predicted to encode mitochondrial proteins [24]. One of them, *NFD3*, encodes S11 mitoRP of the 30S subunit (Table 1). Other Arabidopsis mutants affected in genes that encode mitochondrial proteins such as *gametophytic factor2* [38], *embryo sac development arrest28* (*eda28*) and *eda35* [39] are also defective in cellular nuclei fusion. 

Remarkably, mutations in the plastid ribosomal L21 protein, the only homolog of Arabidopsis NFD1, cause embryonic lethality in the globular stage [40,41]. The different L21 proteins hitherto characterised in several species through the analysis of loss of function mutant alleles, suggest a key role for these proteins in ribosomal function. Nonetheless, their biological effects cannot be directly inferred [40]. Despite being conserved, these proteins might play different complex roles in plant development, partly due to their different subcellular localisation (cytoplasm, mitochondria or chloroplasts). 

### 2.3. Mutations in mitoRP Genes Affect Vegetative Development

#### 2.3.1. Alterations in Leaf Morphology

Defects in leaf development due to mutations in some mitoRPs have been reported: the maize “non-chromosomal stripe” NCS3 mutant displays sectors of poorly developed tissue on leaves and ears, which results from the segregation of somatic wild-type and mutant mitochondria (Table 1) [25]. The molecular nature of this phenotype is a deletion produced by a mitochondrial DNA (mtDNA) rearrangement of a region that contains genes *rps3* and *rpl16*, which respectively code for mitochondrial ribosomal proteins S3 and L16 [25]. Remarkably, Sakamoto et al. [26] also described a mtDNA rearrangement that affects Arabidopsis mitochondrial genes *rps3–rpl16*, caused by the recessive nuclear mutation *chloroplast mutator*, which results in a distorted leaf phenotype (Table 1). Genes *S3* and *L16* have proven to be essential in *E. coli* [42,43] and their protein products appear to function as assembly factors of their corresponding ribosomal subunits [44,45]. More recently, the analysis of the maize *mppr6* mutant impaired in the nuclear gene that encodes mitochondrial pentatricopeptide repeat protein (PPR) MPPR6, which is required for the posttranscriptional regulation of the mitochondrial *rps3* gene, suggests a role of the latter gene also in embryo and endosperm development [46].

Other phenotypes characterised by severe irregularities in leaf morphology have also been reported for defective nuclear genes that encode mitoRPs. Accordingly, the down-regulation by RNAi silencing of the Arabidopsis gene for S10 mitoRP causes severe leaf anomalies (Table 1) [27]. In bacteria, the orthologous protein of S10 is NusE, a multifunctional protein that recruits the ribosome to RNA polymerase [47]. In order to study the effect of S10 mitoRP silencing on vegetative growth, Majewski et al. [27] cultivated transgenic plants under short day conditions (SD) to favour plant growth on reproductive development because SD delays the onset of flowering. Transgenic plants exhibited vastly varying morphologies in relation to the homozygous vs. hemizygous state of the transgene used for gene silencing, and from the timing of its onset [27]. Accordingly, plants homozygous for *S10* silencing, showed severe morphological alterations and some even exhibited small, undulating yellowish leaves that died prior to bolting [27]. 

Kwasniak et al. [48] focused on studying the effects of silencing the Arabidopsis *S10* gene on the expression of the mitochondrial and nuclear genes that encode mitoRPs or proteins of the mitochondrial respiratory chain (Table 1) [48]. They concluded that the perturbation of *S10* alters the levels of the above-mentioned mitochondrial components, especially those encoded by the mitochondrial genome. Thus, in the transgenic plants with the *S10* silenced gene, the transcript levels of the mitochondrial genome genes increased, especially those that code for mitoRPs, whereas those transcribed from the nuclear genes barely alter. At the translational level, mitoRPs and respiratory chain proteins accumulate in the *S10* silenced plants at higher and lower levels than in the wild type, respectively [48]. This suggests the existence of differential changes in mitochondrial translation efficacy when the mitoribosomal function is compromised. The authors proposed that mitoribosomes can self-regulate their own biogenesis by translational control, as previously reported in bacteria and chloroplasts [49,50]. The results of Kwasniak et al. [48] support the ribosomal filter hypothesis proposed by Mauro and Edelman [22], which states that ribosomes are not simple machines for mRNA translation, but can act as regulators of gene expression by acting as a filter that differentially affects the translation of different transcripts. In line with this, defective mitoribosomes, due to the silencing of the S10 protein, would differentially affect the translation of different mRNA species.

Consistent with this view, Schippers and Mueller-Roeber [21] have reported that the expression of the genes that encode mitoRPs and the relative translational activity of different ribosomal protein transcripts in several leaf tissues are highly variable during leaf development in Arabidopsis.

#### 2.3.2. Mutations in mitoRPs and the OGE Retrograde Signalling Pathway

Other mutations in *mitoRP* genes have a subtle effect on leaf development. For instance, the Arabidopsis mutant defective for the nuclear gene that encodes L11 mitoRP shows reduced mitochondrial respiratory proteins abundance, which suggests an alteration in mitochondrial activity. As a likely consequence, *mrpl11* plants display stunted plant size and a darker leaf colouring than the wild type (Table 1). However, no clear alteration in leaf morphology has been reported [28]. In *E. coli*, L11 is a non-essential protein [42] and constitutes one of the main anatomical features of the 50S ribosomal subunit, the L11 arm, which includes the binding site for the 23S rRNA [51], and may be important for translation termination [52]. Pesaresi et al. [53] had previously reported that the *prpl11* mutant, which is affected in the nuclear gene that encodes the plastid L11 protein, shows reduced growth and pale pigmentation in cotyledons and leaves. Interestingly, double mutant plants *mrpl11 prpl11*, but none of the single mutant plants, display a drastically reduced expression of nuclear genes that encode photosynthetic proteins targeted to chloroplasts (Table 1) [28]. The repression of nuclear photosynthetic genes may result from perturbed plastid and/or mitochondrial gene expressions due to the activation of the retrograde signalling pathway named OGE (organelle gene expression). Therefore, the results reported by Pesaresi et al. [28] indicate cooperation for the signals emitted by chloroplasts and mitochondria to regulate the expression of nuclear photosynthetic genes when translation in both organelles is disturbed. This down-regulation of nuclear photosynthetic genes is similar to that reported for the Arabidopsis *prors1-1* and *1-2* mutants affected in the nuclear gene that encodes the prolyl-tRNA synthetase protein targeted to both chloroplasts and mitochondria [28]. Remarkably, null mutant alleles *prors1-3* and *1-4* are embryonic-lethal as they arrest embryonic sac formation and, hence, embryo development [28].

## 3. Defective Mitoribosomal Function by Mutations in Mitochondrial Proteins Other than mitoRPs

The plant mitoribosome function can be modulated by the activity of nuclear genes that encode mitochondrial-targeted proteins apart from mitoRP. One example of this is the PPR family of proteins, a large group of eukaryotic-specific modular RNA proteins encoded by the nucleus that have undergone expansion in terrestrial plants [54]. PPR proteins are important for the expression of organelle genomes and organelle biogenesis because they are involved in transcription, and also in RNA stability, processing, splicing, editing and translation [54,55]. In line with this, the Arabidopsis PPR336 protein has been associated with mitochondrial polysomes and is required for the stability of mitoribosomes [54]. Notwithstanding, no morphological alterations have been described for the mutants affected in the *PPR336* gene. Despite this, the mitochondrial polysomes in these mutant plants have a lighter molecular weight than those of wild-type plants, which might have an effect on mitochondria protein translation [56]. Interestingly, Del Valle-Echevarria et al. [57] found that the MCS16 mosaic mutant of cucumber, which displays distorted cotyledons, chlorotic leaves, stunted growth and reduced fertility, also shows lower levels of the transcripts of the *rps7* mitochondrial gene, which codes for S7 mitoRP. These authors proposed the *PPR336* gene of cucumber to be the likely candidate responsible for the phenotype of the MCS16 mutant as PPR336 is required for the accurate processing of *rps7* transcripts [58]. The S7 protein is essential in *E. coli* [42,43] and, together with the S11 protein, forms the 30S E (exit) site [59]. Besides, S7 binds to 16S rRNA and functions as an assembly initiator of the 30S subunit in bacteria [60].

In Arabidopsis, another PPR protein, the product of the *PNM1* (*PPR protein localized to the nucleus and mitochondria 1*) gene, has also been reported to be associated with mitochondrial polysomes in an RNA-dependent manner [59]. Remarkably, impaired *PNM1* function in the mitochondria is embryo-lethal, although it has not been possible to identify the precise RNA targets of the PNM1 protein [61]. The null mutations in the *EMP5* gene (*EMPTY PERICARP5*) of maize, which encodes a DYW subgroup of PPR proteins involved in editing several mitochondrial transcripts, result in kernels devoid of embryo or endosperm structures, which reveals a role for this gene in seed development [62]. Interestingly, these defects are due mainly to the incorrect editing of *rpl16* mitochondrial transcripts by changing a leucine for a proline residue at position 153. This change may be critical for the L16 protein function, and hence for mitorribosome activity, as it alters organelle function and compromises seed development. This would extend the *rpl16* function to not only leaf morphogenesis, as previously mentioned (see Section 2.3.1), but also to seed development. The *EMP5* function seems conserved in rice because its down-regulation results in defective seeds and slower seedling growth, which indicates other roles for this protein in plant development apart from embryonic ones. Remarkably, the Arabidopsis *mef35* (*mitochondrial editing factor 35*) mutant, which is affected in a nuclear gene encoding, as *EMP5*, a DYW PPR protein, also displays a defect in the editing of the mitochondrial *rpl16* transcript by changing a very conserved threonine of the L16 protein for isoleucine [63]. Yet unlike *emp5*, this change has no phenotypic effects on *mef35* plants and questions whether the edition of *L16* mediated by MEF35 has any functional consequences.

## 4. Conclusions and Future Perspectives

**Figure 1:**
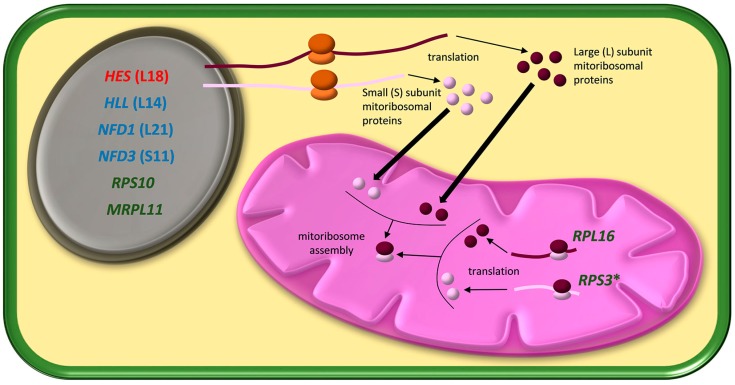
Genes that encode mitochondrial ribosomal proteins (mitoRPs) whose mutations cause developmental defects are shown in the diagrams for the nucleus (grey) and mitochondria (magenta). The mRNAs encoding proteins of the large (dark purple spheres) and small (light purple spheres) subunits are shown in dark purple and light purple, respectively. The genes characterised from the analysis of the mutants defective in embryonic, vegetative or reproductive development are respectively depicted in red, blue and green. When a gene was named according to a mutant phenotype, the encoded mitoRP is shown in parentheses. Cytosolic ribosomes are depicted in orange and mitorribosomes in purple. *HES*: *HEART STOPPER*; *HLL*: *HUELLENLOS*; *NFD1* and *3*: *NUCLEAR FUSION DEFECTIVE 1* and *3*. * The mutations that affect the genes in this figure were all characterised in *Arabidopsis thaliana*, except for *RPS3*, for which a mutant allele was also described in *Zea mays*.

In plants, only a few mutants affected in mitoRPs have been hitherto described and characterised phenotypically and molecularly. Therefore, information on the contribution of plant mitoRPs and, by extension, mitoribosomes, to plant growth and to different development stages is still scarce. Nevertheless, the results obtained in recent years by characterising several plant mutants defective in mitoRPs reveals a prominent role for these proteins in plant morphogenesis (Figure 1). Some of the results obtained to date support the participation of specific mitoRPs in different developmental processes, which might be interpreted as a result of the functional specialisation of distinct mitoRPs [20,23,24,25,26,27,28]. Accordingly, the modification of the protein composition of mitoribosomes in various plant tissues, organs or developmental stages may be a mechanism to help regulate its activity and, finally, the expression of the genes whose products are located in mitochondria. Consequently, mitochondrial activity would adjust to the needs of the biological processes that take place at specific times of development. If this were the case, it would support the plant mitoribosomes heterogeneity hypothesis, which is the basis of the so-called ribosomal filter hypothesis [22]. In this review, we focused on several pieces of genetic evidence that support this hypothesis in plant mitochondria. To strengthen such evidence, we consider it necessary to look in-depth into the isolation and characterisation of new mutants affected in mitoRP genes in Arabidopsis and other plant species. Special attention should be paid to the mutants defective in different members of gene families to identify differential phenotypic effects. In a plant model such as Arabidopsis, it is possible to screen collections of insertional mutations, mainly induced by T-DNA, to cover almost every gene [64]. This allows systematic screening for those mutants defective in each predicted *mitoRP* gene. Nonetheless, some genes may not be tagged and, even if they are, the insertion might not affect the function of the corresponding protein or cause a desirable structural or functional change. New genome editing tools based on the CRISPR/Cas system could overcome these limitations [65] and be used to generate new alleles of either previously described nuclear *mitoRP* genes or novel ones. In line with this, a mitochondria-targeted Cas9 (mitoCas9) protein has been designed and used in cultured human cells to edit the mitochondrial genome [66]. Gene editing might also be applied to create a series of hypomorphic alleles of mitoRP genes. To date, only null alleles of the *HES*, *HLL* and *NFD* genes causing embryonic, ovule or gametophyte lethality respectively, have already been described [20,23,24]. Therefore, the identification and characterisation of hypomorphic alleles of these genes should be instrumental to ascertain if the functions of the corresponding mitoRPs are restricted exclusively to early development. To define the post-embryonic functions of lethal genes, other genetic and molecular strategies, such as clonal analysis in post-embryonic tissues [67], lethality rescue based on inducible promoters [68] or post-embryonic knock-down mediated by tissue-specific [69] or inducible promoters [70], may also be used.

Besides genetic evidence, it has been proposed that demonstration of the existence of specialized ribosomes will require resolving three main challenges: (a) the isolation of naturally-occurring specific homogenous ribosomes; (b) their structural, biochemical, molecular and cellular characterisation; (c) the identification and validation of the different substrates of the specialised ribosomes [71]. A plethora of new technical advances, such as single-particle cryo-electron microscopy [72] and serial femtosecond X-ray crystallography [73], among others, might contribute to characterise the different ribosomes found in a particular species, organ, tissue, cell or organelle, and to set up their unique structural and functional properties. This is particularly relevant in plants because to date, the cryo-EM structure of mitorribosomes is still lacking. A recent study into cytosolic ribosomes of mouse embryonic stem cells by quantitative mass spectrometry has revealed a functional link between ribosome heterogeneity, at the RPs composition level, and gene regulation [74]. Consequently, translating ribosomes lacking particular RPs associate with specific types of mRNAs. Similar studies of organelle ribosomes are expected to also reveal a functional relationship between its composition and the control of the gene expression in mitochondria and chloroplasts.

## Figures and Tables

**Table 1:** Plant mitochondrial ribosomal proteins characterized from the analysis of developmental mutants.

Defects in	Gene	mitoRP ^a^	Species	Mutant Phenotype
*Embryo development*	*HEART STOPPER* (*HES*) ^b^ AT1G08845 ^d^	L18	*Arabidopsis thaliana*	Reduced proliferation of endosperm cells and arrested embryo development in the late globular stage [20]
*Reproductive development*	*HUELLENLOS* (*HLL*) ^b^ AT1G17560 ^d^	L14	*Arabidopsis thaliana*	Early cellular degeneration of the eggs, characterised by arrested ovule development before or just after the formation of the integuments (*hll-1*) or after the integuments have begun to spread around the nucela (*hll-2*). *hll-1* and *hll-2* also show alterations in the gynoecium [23]
	*NUCLEAR FUSION DEFECTIVE1* (*NFD1*) ^b^ AT4G30925 ^d^	L21	*Arabidopsis thaliana*	Defective in kariogamy during fertilization and development of the female and male gametophytes [24]
	*NFD3* ^b^ AT1G31817 ^d^	S11	*Arabidopsis thaliana*	Defective in kariogamy during fertilization and development of the female gametophyte [24]
*Vegetative development*	*rps3* ^c^ and *rpl16* ^c^	S3 and L16	*Zea mays*	Sectors of poorly developed tissue on leaves and ears, which result from the segregation of somatic wild-type and mutant mitochondria [25]
	*rps3* ^c^ and *rpl16* ^c^ AtMg00090 ^d^ and AtMg00080 ^d^	S3 and L16	*Arabidopsis thaliana*	Distorted leaf phenotype [26]
	*Rps10* ^b^ AT3G22300 ^d^	S10	*Arabidopsis thaliana*	Plants homozygous for S10 silencing, show severe morphological alterations; they exhibit small, undulating, and yellowish leaves and died prior bolting [27]
	*Mrpl11* ^b^ AT4G35490 ^d^	L11	*Arabidopsis thaliana*	Stunted plant size and a darker leaf coloring than the wild type [28]

^a^ Mitochondrial ribosomal protein; ^b^ Nuclear gene; ^c^ Mitochondrial gene; ^d^ AGI code.
